# The institutionalized elderly: sociodemographic and clinical-functional profiles related to dizziness^☆^^☆☆^

**DOI:** 10.1016/j.bjorl.2014.12.014

**Published:** 2015-12-11

**Authors:** Tábada Samantha Marques Rosa, Anaelena Bragança de Moraes, Valdete Alves Valentins dos Santos Filha

**Affiliations:** a Postgraduate Program in Human Communication Disorders (PPGDCH), Universidade Federal de Santa Maria (UFSM), Santa Maria, RS, Brazil; b Department of Statistics, Universidade Federal de Santa Maria (UFSM), Santa Maria, RS, Brazil; c Epidemiology, Universidade Federal do Rio Grande do Sul (UFRGS), Porto Alegre, RS, Brazil; d Speech Therapy Course, Universidade Federal de Santa Maria (UFSM), Santa Maria, RS, Brazil; e Postgraduate Program in Communication Sciences – Human Communication, Universidade Federal de Santa Maria (UFSM), Santa Maria, RS, Brazil

**Keywords:** Elderly, Long-stay institution for the elderly, Health profile, Dizziness, Idoso, Instituição de longa permanência para idosos, Perfil de saúde, Tontura

## Abstract

**Introduction:**

Dizziness is among the most common complaints in the elderly population.

**Objective:**

To determine the sociodemographic and clinical-functional profiles of institutionalized elderly people related to dizziness.

**Methods:**

Cross-sectional prospective study with institutionalized elderly people aged 60 or more years. A questionnaire on sociodemographic and clinical-functional characteristics was applied, and an anamnesis of occurrence of dizziness was held, as well as the Dizziness Handicap Inventory questionnaire.

**Results:**

48.9% of the elderly subjects had dizziness. The mean numbers of diseases and medications associated with dizziness were, respectively, 4.5 diseases and 7.8 medications. We found a significant association between the occurrence of dizziness and diseases of the musculoskeletal system, sub-connective tissue and genitourinary system, as well as the use of medications for the musculoskeletal system. The scores for handicap degree in functional DHI were significantly higher among elderly subjects who needed walking aids, who had suffered falls, and those manifesting anxiety.

**Conclusion:**

Our sample included subjects of advanced age, primarily women, who were institutionalized less than five years, with multiple diseases and polypharmacy users. They presented long-standing short-duration mixed dizziness, that occurred more than once a month and affected mainly the functional aspect.

## Resumo

**Introdução:**

A tontura está entre as queixas mais comuns da população idosa.

**Objetivo:**

Determinar os perfis sociodemográfico e clínico-funcional de idosos institucionalizados com relação à tontura.

**Método:**

Estudo prospectivo transversal, com idosos institucionalizados com ≥ 60 anos de idade. Foi aplicado um questionário referente às características sociodemográficas e clínico-funcionais, assim como foi feita anamnese sobre a ocorrência de tontura, e aplicado o questionário *Dizziness Handicap Inventory*.

**Resultados:**

48,9% dos idosos apresentaram tontura. As médias do número de doenças e medicamentos associados à tontura foram, respectivamente, 4,5 doenças e 7,8 medicamentos. Houve associação significativa entre ocorrência de tontura e doenças do sistema osteomuscular, do tecido subconjuntivo e do aparelho geniturinário, bem como uso de medicamentos para o sistema musculoesquelético. Os escores do grau de handicap no DHI funcional foram significativamente maiores para os idosos que necessitavam de auxílio à marcha, para os que tiveram queda e para os que apresentaram ansiedade.

**Conclusão:**

Amostra caracterizada por mulheres de idade elevada, com menos de cinco anos de institucionalização, com múltiplas doenças e medicamentos. Apresentam tontura mista, de curta duração, com surgimento há anos, manifestando-se mais de uma vez ao mês, prejudicando principalmente o aspecto funcional.

## Introduction

Human aging compromises certain skills of the central nervous system (CNS), and affects areas responsible for signal processing from the vestibular, visual and proprioceptive systems. These sensory systems are essential for maintaining body balance and when affected, negatively impact the ability to change adaptive reflexes,^1^ responsible for postural control and orientation of the body relative to space.^2^

Dizziness is among the most common complaints in the elderly and may be characterized as a sense of giddiness, a feeling of “light-headedness”, a sense of imminent fall, instability, a floating sensation, vertigo, a tendency to deviate when walking, body imbalance, falls, and spatial disorientation, among others.^3^ These changes may result in fractures, loss of mobility and dependence on others to perform daily activities.^4^

In the world's population, 10–15% of people have dizziness, and this complaint ranks seventh place among those most frequently found in women, and is the fourth most common complaint among men. After 65 years, balance changes are considered to be the most common symptoms in the geriatric population, reaching a prevalence of 85%.^5^ Three out of four Americans aged 70 or older have postural balance problems.^6^ The causes of dizziness may be associated with organic and/or psychic dysfunction, which have extra-vestibular (visual, neurological, emotional) or vestibular origin.^7^, ^8^

In order to quantify interferences from physical and functional/emotional dizziness on daily activities of a subject suffering vertigo, a specific questionnaire called Dizziness Handicap Inventory (DHI) was developed and validated by Jacobson and Newman,^9^ with the aim of evaluating the self-perception of the incapacitating effects caused by dizziness.^10^ In a study in which DHI was applied, all elderly subjects tested showed changes in quality of life; physical aspects were the most affected, and functional aspects were the most affected in older individuals.^11^

People with dizziness usually report difficulty with mental concentration, memory loss and fatigue. Besides generating physical insecurity, these symptoms can lead to psychic insecurity, irritability, loss of self-confidence, anxiety, depression, or panic.^12^

Also involved in dizziness in addition to the factors mentioned above, a large number of the elderly population take multiple medicines (polypharmacy) because they also are afflicted with a high number of comorbidities that bring about physiological changes in their pharmacokinetics and pharmacodynamics; this adds to the ongoing degenerative process.^13^

The number of drugs used by the elderly population is a major risk factor for iatrogenic events, with an exponential relationship between magnitude of polypharmacy and the likelihood of adverse reactions, drug interactions and the use of inappropriate drugs.^14^ Thus, elderly people residing in long-stay institutions for the elderly (LSIEs) are at increased risk for iatrogenic events, due to disabling diseases, frailty and low functionality,^15^ sometimes requiring drug prescription.

The use of tools to investigate the clinical-functional status of elderly people with and without body balance disorders, and the relationship to sociodemographic characteristics, can add information to assist the search for a timely diagnosis and a more effective therapeutic orientation.

Thus, the aim of this study is to determine the socio-demographic and clinical-functional profiles of institutionalized elderly subjects related to dizziness.

## Methods

This is a cross-sectional historical cohort study, conducted in three philanthropic long-stay institutions for the elderly from August 2013 to January 2014. The study was approved by the Research Ethics Committee under opinion number 322,139 on its 20 June 2013 session. In order to obtain a Free and Informed Consent Term (FICT), we complied with the recommendations from Resolution number 466/2012 of the National Council of Health, which regulates research involving human beings.

The subjects signed the FICT form after reading it and with their concerns and questions already clarified by the researcher. For those presenting some motor and/or visual disability that prevented them from writing, the form was signed by the head of the institution.

For sample selection, the following inclusion criteria were considered: individuals aged ≥60 years, according to Decree number 1,395/GM of the National Health Policy for the Elderly,^16^ residing in a charitable long-stay home. Individuals presenting neurological disorders; judgment, language and/or cognition loss, or dementia, who were not being able to understand the necessary procedures for the assessment of study variables, would not agree to take part in the study or sign the FICT form, were excluded from this study.

This was a consecutive, convenience sample totaling 142 elderly subjects. Of these, 44 were excluded because they fell below the minimum cutoff level of 10 points in the evaluation of the Mini-Mental State Examination (MMSE) measuring the ability of understanding and verbal communication, according to Folstein, Folstein and McHugh.^17^ Thus, the final sample consisted of 98 elderly individuals; 32 were male and 66 female.

Because of the limited information on education in the medical records of the elderly subjects, and considering that the classification of Folstein, Folstein and McHugh^17^ presents large score ranges for the categorization of dementia, we decided in favor of that classification in this study. The evaluations of the elderly subjects were held individually in a room made available for these activities in each LSIE. A meeting with the head of each institution to discuss the operational organization of this research, had been completed previously.

At first, the elderly were evaluated through a questionnaire composed of open and closed questions during a face-to-face interview. This instrument had been previously tested with 30 seniors, and the necessary corrections were made. For elderly subjects presenting difficulty of expression of spoken language, the assistance of their caregivers was requested, and for those who showed fatigue during the interview an additional interview was scheduled.

The study variables were classified as socio-demographic (gender, age, skin color, marital status, education and time since being institutionalized), which were obtained directly from medical records, and clinical-functional. The clinico-functional group included the number and type of diseases and drugs, classified according to the International Classification of Diseases (ICD-10) and the Anatomical Therapeutic Chemical (ATC) Classification Index, the use of walking aids, the occurrence of falls, fractures in upper and lower limbs related to falls, a subjective perception of vision and hearing (very poor, poor, fair, good, or excellent), the frequency of physical activity and emotional issues such as presence or absence of anxiety and depression, and whether the subject was undergoing psychological and/or psychiatric treatment.

It should be noted that the diagnosis of diseases and prescribed drugs was obtained directly from the medical records of each elderly subject, for which the LSIE's doctors were responsible.

Subsequently, a history was obtained concerning the occurrence of dizziness, and information about its onset, type, triggering factors, intensity and duration, frequency, interference with daily activities, and any neurodegenerative manifestations, habits and subjective perception of dizziness, as, classified according to the Visual Analogue Scale (VAS), was registered.

Finally, the DHI questionnaire, developed by Jacobson and Newman^9^ and containing 25 questions assessing self-perception of the incapacitating effects caused by dizziness (emotional, physical and functional aspects), was applied. This questionnaire was adapted to the reality of institutionalized elderly persons. To analyze the results, three possible answers were considered in the questionnaire, with the following scores: yes = 4 points; sometimes = 2 points, and no = 0 points. The maximum possible scores in emotional, physical, and functional domains were 32, 36, and 32 points, respectively, totaling a possible maximum score of 100 points. For the total DHI score, the higher the value, the higher the degree of disability of the elderly subject in the context of dizziness.

To analyze statistical data, we initially conducted a descriptive analysis and, later, an inferential analysis using non-parametric tests: chi-square, Fisher exact, Mann–Whitney *U*, Kruskal–Wallis, and post hoc; the level of significance was set at 5%. Analyses were performed using the STATISTICA 9.1 software.

## Results

Our sample consisted of 98 institutionalized elderly subjects, with a mean age of 76.3 years (±8.5), ranging from 60 to 94 years. The mean time of institutionalization was 5.6 years (±6.9), ranging from 2.4 months to 42 years. With respect to dizziness complaints (*n* = 48), 48.9% reported that they experienced dizziness. There was no significant association of dizziness with the socio-demographic variables considered.

Table 1 lists the distribution of elderly subjects according to socio-demographic variables, and their association with the occurrence of dizziness.

**Table 1:** Distribution of frequencies of institutionalized elderly people with respect to sociodemographic aspects and dizziness (*n* = 98).

Variable	Categories	Number of elderly subjects (%)	Number of elderly subjects with dizziness (%)	*p*-value
Gender	Male	32 (32.7)	14 (29.2)	0.471
	Female	66 (67.3)	34 (70.8)	

Skin color	Caucasian	77 (78.6)	39 (81.2)	0.703
	Black	15 (15.3)	7 (14.6)	
	Mixed-race	6 (6.1)	2 (4.2)	

Marital status	Single	50 (51.0)	25 (52.0)	0.506
	Married	2 (2.0)	2 (4.2)	
	Separated	3 (3.1)	1 (2.1)	
	Widowed	39 (39.8)	19 (39.6)	
	Divorced	4 (4.1)	1 (2.1)	

Schooling^a^	Illiterate	14 (14.3)	6 (12.5)	0.840
	Incomplete elementary school	73 (74.5)	36 (75.0)	
	Complete elementary or more	11 (11.2)	6 (12.5)	

Age (years)	60–70	25 (25.5)	9 (18.7)	0.245
	71–80	42 (42.9)	24 (50.0)	
	≥81	31 (31.6)	15 (31.3)	

Time since being institutionalized (years)	0.1–4.9	55 (56.1)	31 (64.6)	0.155
	5–9.9	29 (29.6)	13 (27.1)	
	≥10	14 (14.3)	4 (8.3)	

Total		98 (100.0)	48 (100.0)	

Chi-square test, or Fisher's exact test.

a Incomplete elementary education, 1–4 years of schooling; complete elementary education or more, ≥5 years of schooling.

Table 2 shows data on the prevalence of diseases among the elderly, and associations with the occurrence of dizziness.

**Table 2:** Clinical-functional characteristics of institutionalized elderly people and association between the types and number of diseases and occurrence of dizziness (*n* = 98).

Types of diseases	Number of elderly subjects (%)	Number of elderly subjects with dizziness (%)	*p*-value
*Infectious and parasitary*	5 (5.1)	1 (2.1)	0.362
*Neoplasias (tumors)*	5 (5.1)	4 (8.3)	0.200
*Blood*	5 (5.1)	4 (8.3)	0.200
*Endocrine*	53 (54.0)	23 (47.9)	0.311
*Mental disorders*	41 (41.8)	21 (43.8)	0.838
*Nervous system*	91 (92.8)	43 (89.6)	0.264
*Eyes and adnexa*	25 (25.5)	12 (25.0)	1.000
*Circulatory system*	71 (72.4)	32 (66.7)	0.260
*Respiratory system*	9 (9.1)	2 (4.2)	0.160
*Digestive tract*	61 (62.2)	34 (70.8)	0.099
*Musculoskeletal system*	55 (56.1)	33 (68.8)	0.016^a^
*Genitourinary tract*	29 (29.5)	19 (39.6)	0.046^a^

*Number of diseases*			
≤2	11 (11.2)	3 (6.2)	0.200
≥3	87 (88.8)	45 (93.8)	

Blood, blood and hematopoietic organs, and immune disorders; Endocrine, nutritional endocrine and metabolic diseases; mental disorders, mental and behavioral disorders. Chi-square or Fisher's exact test.

a Significant association (*p* ≤ 0.05).

Table 3 lists data on the frequency distribution of the types of drugs used, as well as associations with the occurrence of dizziness.

**Table 3:** Clinical-functional characteristics of institutionalized elderly people and the association between types and number of drugs and the occurrence of dizziness (*n* = 98).

Type of drug	Number of elderly subjects (%)	Number of elderly subjects with dizziness (%)	*p*-value
*Otoneurological*	20 (20.4)	12 (25.0)	0.321
*Cardiovascular system*	67 (68.3)	30 (62.5)	0.279
*Digestive tract*	82 (83.6)	43 (89.6)	0.172
*Nervous system*	88 (89.7)	42 (87.5)	0.520
*Hematological system*	1 (1.0)	1 (2.1)	0.490
*Muscular system*	55 (56.1)	33 (68.8)	0.016^a^
*Respiratory system*	8 (8.1)	1 (2.1)	0.060
*Hormonal preparations*	55 (56.1)	24 (50.0)	0.309
*Ophthalmological*	24 (24.4)	12 (25.0)	1.000

*Number of drugs*^a^			
1 or 2	7 (7.1)	3 (6.3)	0.506
3 or 4	14 (14.3)	5 (10.4)	
≥5	77 (78.3)	40 (83.3)	

Muscular system, musculoskeletal system and sub-connective tissue; digestive tract, digestive and metabolic tract; hormonal preparations, systemic hormonal preparations. Chi-square or Fisher's exact test.

a Significant association (*p* ≤ 0.05).

The mean number of associated diseases per institutionalized elderly subject was 4.5 (±1.6), with a maximum of 8; 10 elderly patients (10.2%) presented 1 or 2 diseases, 39 (39.7%) had 3 or 4 diseases, and 48 (48.9%) had ≥5 diseases. As to the number of medications used, we found 7.8 (±3.7) drugs per elderly subject, with a maximum of 17 drugs. All the elderly subjects in this study were taking some kind of medication.

Table 4 shows the prevalence of other clinical-functional characteristics: use of walking aids, occurrence of falls, physical activity, anxiety, depression, and the association of these variables with the occurrence of dizziness.

**Table 4:** Clinical-functional characteristics of institutionalized elderly people and association with occurrence of dizziness (*n* = 98).

Variables	Categories	Number of elderly subjects (%)	Number of elderly subjects with dizziness (%)	*p*-value
Walking aid	Yes	54 (55.2)	30 (62.5)	0.149
	No	44 (44.8)	18 (37.5)	

Falls^a^	Yes	73 (74.5)	39 (81.2)	0.133
	No	25 (25.5)	9 (18.8)	

Physical activity	Does not practice	6 (6.1)	2 (4.2)	0.117
	Every day	25 (25.6)	8 (16.7)	
	Weekly	32 (32.6)	20 (41.6)	
	Biweekly or more	35 (35.7)	18 (37.5)	

Anxiety	Yes	85 (86.8)	42 (87.5)	0.213
	No	13 (13.2)	6 (12.5)	

Depression	Yes	80 (81.7)	43 (89.6)	0.080
	No	18 (18.3)	5 (10.4)	

Chi-square test or Fisher exact test for association.

a During the institutionalization period.

Although it has been shown that the occurrence of falls and presence of anxiety and depression have no significant association with dizziness, our findings demonstrate important numerical differences with regard to complaints of dizziness among elderly people with or without such morbidities.

Historical data revealed that 29.2% of elderly patients with dizziness had suffered fractures due to falls, but a significant association (*p* = 0.562) was not found. As for self-perception of sight and hearing, a high number of subjects chose the “normal” option for both sight (40.8%) and hearing (36.7%). Considering only those who experienced dizziness, the “poor” option was chosen for both sight (35.4%) and hearing (37.5%). We must emphasize that, considering the group of elderly patients with dizziness, 60.4% are or were in psychological treatment, and 16.6% are or were receiving psychiatric treatment.

Table 5 shows the characteristics of dizziness (onset, type, triggering factors, duration and frequency).

**Table 5:** Features of dizziness in institutionalized elderly subjects (*n* = 48).

Variable	Categories	Number of elderly subjects with dizziness (%)
Onset	Days	4 (8.3)
	Weeks	3 (6.2)
	Months	13 (27.0)
	Years	28 (58.5)

Type	Rotational	20 (41.6)
	Instability	5 (10.4)
	Imbalance	26 (54.1)
	Fall	1 (2.0)

Triggering factors	Turn the head from side to side	19 (39.7)
	Quickly lift the trunk	17 (35.4)
	Stand up fast from a sitting position	10 (20.8)
	Extend the head backwards	2 (4.1)

Duration	Long duration	21 (42.6)
	Short duration	27 (57.4)

Frequency	Every day	17 (35.4)
	More than once a month	26 (54.2)
	Less than once a month	5 (10.4)

*Note*: The sum of the number of elderly subjects may exceed 48, because of possible involvement in more than one category.

Regarding self-perception of the intensity of dizziness by the elderly as measured by EVA, a mean of 6.2 points (±1.1) (range: 3–8 points) was obtained. There was no significant difference (*p* = 0.897) for self-perception values on the intensity of dizziness in the age groups. However, in relation to gender, men showed significantly higher values for intensity of dizziness *compared to* women (*p* = 0.012).

Considering only those elderly individuals who complained of dizziness, we identified that the most compromised daily activity was physical activity (87.5%). Neuro-vegetative symptoms were identified in 52.1% of the elderly; the symptoms most commonly reported were nausea (29.2%), tachycardia (18.8%), sweating and diarrhea (4.2%). Among the habits of the elderly with dizziness, coffee consumption was the most frequently mentioned (87.5%), followed by the consumption of “chimarrão” (a type of tea) (58.3%), sugar (56.3%), and tobacco (6.3%).

Table 6 lists a comparison of the scores for DHI subscales with respect to the use of walking aids, occurrence of falls, physical activity, anxiety and depression, as well as descriptive measures of total DHI scores by gender, age, time since being institutionalized and the significance in the comparison of these scores. Although the occurrence of depression, either previous or current, was not significantly associated with dizziness, a higher percentage of dizziness in the elderly currently depressed was found.

**Table 6:** Comparison between scores of DHI subscales and total DHI score versus clinical-functional and sociodemographic aspects of institutionalized elderly people (*n* = 48).

Variables	DHI (*p*-value)			
Clinical-functional	Physical	Functional	Emotional	Total
Walking aid	0.416	0.013^a^	0.295	0.046^a^
Falls	0.567	0.046^a^	0.219	0.081
Physical activity	0.073	0.643	0.129	0.344
Anxiety	0.820	0.042^a^	0.103	0.201
Depression	0.392	0.073	0.219	0.141
Median of scores (min–max)	12 (0–26)	12 (0–26)	17 (0–34)	40 (2–80)

Sociodemographic features	Total DHI			
	(min–max)	Q1; Median; Q3	Mean ± SD	*p*-value
*Gender*				
Male (*n* = 14)	20.0–66.0	34.0; 41.0; 52.0	42.6 ± 12.1	0.641
Female (*n* = 34)	2.0–80.0	32.0; 40.0; 50.0	39.5 ± 18.6	

*Age* (years)				
60–70 (*n* = 9)	6.0–56.0	32.0; 40.0; 48.0	37.3 ± 16.0	0.728
71–80 (*n* = 24)	2.0–68.0	31.0; 40.0; 50.0	39.4 ± 16.7	
81 or more (*n* = 15)	4.0–80.0	32.0; 44.0; 56.0	43.8 ± 18.2	

*Institutionalization time* (years)				
0–4.9 (*n* = 31)	2.0–80.0	32.0; 42.0; 56.0	41.2 ± 18.2	0.823
5–9.9 (*n* = 13)	2.0–66.0	32.0; 40.0; 50.0	38.4 ± 15.9	
≥10 (*n* = 4)	28.0–52.0	33.0; 40.0; 47.0	40.0 ± 9.9	

DHI, Dizziness Handicap Inventory; Q_1_, 1st quartile; Q_3_, 3rd quartile; SD, standard deviation.

Mann–Whitney U or Kruskal–Wallis tests.

a Statistical significance for *p* ≤ 0.05.

With respect to functional DHI, statistical values were significantly higher for those participants who needed some help to walk, for those who suffered falls, and for those presenting in an anxiety state. In the comparison of total DHI scores, according to socio-demographic variables (gender, age, and time since being institutionalized), no significant differences were found, but we did identify a direct proportional relationship between the age of the elderly and total DHI score.

## Discussion

Over the years, the sensory and motor systems responsible for maintenance of equilibrium suffer degenerative, infectious and traumatic processes that hinder their optimal functioning.^18^

Although there was no significant association with those socio-demographic characteristics we considered with respect to the importance of the prevalence of dizziness in the elderly, we found that a great number of institutionalized elderly persons (48.9%) complained of dizziness; this is similar to the study by Borges, Garcia and Ribeiro,^19^ who identified a prevalence of 58.0% for dizziness complaints in their group of institutionalized elderly persons. In other studies with community-based older adults, the prevalence of dizziness was reported to be 42% and 45%.^20^, ^21^

In the present study, 70.8% of the elderly people with dizziness, were female, which is in line with studies in the literature that found 53%^20^ and 80%^22^ of female patients among elderly people with vestibular disorders. This could be explained by a greater concern on the part of women to seek medical advice, compared to men,^20^, ^22^ and also by the fact that, with menopause, women are more prone to heart disease, suggesting a strict relationship between blood pressure and female hormones.^23^ With regard to skin color, most elderly patients with dizziness (81.2%) were Caucasian, though this lacked statistical significance. No studies were found establishing an association of skin color or ethnic data with the occurrence of dizziness in the elderly. In studies published, white skin color was present in 67.0%,^24^ 80.5%,^25^ and 90.0%^26^ of institutionalized elderly persons, similar to our study (78.6% of older people with white skin color).

As to marital status, most elderly subjects were single (51.0%). Other studies have found 38.9%^27^ and 63.0%^24^ of single elderly subjects. No studies were found analyzing dizziness associated with marital status in this population.

In this study, the level of education “completed elementary or more” occurred in 12.5% of elderly patients with dizziness; most subjects with such symptom (75%) stated an “incomplete elementary education”; this association lacked statistical significance. In the reviewed literature, 17.5%^28^ and 30.8%^21^ of elderly patients with balance disorders had “completed elementary education or more.”

A greater level of education is thought to exert a positive influence in maintaining brain structures and increasing synaptic density, which helps to reduce the damage suffered by the CNS with aging.^29^

In this study, the majority of institutionalized elderly subjects were in the 71–80 years range, with a mean age of 76.3 years, similar to the study by Borges, Garcia and Ribeiro^19^ that found a mean of 74.6 years for institutionalized elderly people. In another longitudinal study, 27.0% of non-institutionalized elderly subjects aged > 65 years complained of dizziness during the preceding six months, and 54.0% of those aged > 70 years reported it,^30^ which is in line with our findings: 57.1% prevalence for elderly subjects aged 71–80 years.

Our subjects had a mean of 5 years and 6 months since being institutionalized, affirming data from a study that reported 5 years and 8 months.^31^ It is believed that the institutionalization process occurs because the elderly do not have adequate physical, and psycho-social, conditions to live alone, and sometimes they do not have family support from their children or spouses.^24^

In the analysis of clinical-functional characteristics, we identified a statistically significant difference between the occurrence of dizziness and diseases of the musculoskeletal system, sub-connective tissue and genitourinary system, with a prevalence of 56.1% and 39.6%, respectively. Despite the lack of a significant association, diseases of the nervous system have been identified as the most prevalent characteristics (89.6%). In the literature, lower percentages were identified for diseases of the musculoskeletal system and sub-connective tissue (41.2%),^32^ genitourinary tract (12.5%)^28^ and nervous system (10%).^28^

It is important to note that changes in the osteo-articular system such as physiological osteopenia, cartilaginous aging, sarcopenia and reduction in nerve conduction velocity are strongly associated with the aging process. Pain and disorders of the musculoskeletal system are the most frequent complaints of the elderly, and rheumatic diseases also had a higher incidence with advancing age, such as osteoporosis, osteoarthritis, and polymyalgia rheumatica.^33^ In the same context, researchers state that diseases of the circulatory and musculoskeletal systems, as a result of peripheral and/or central involvement, can manifest themselves in the form of balance disorders and causes of instability.^2^

According to the literature, despite the lack of neurological symptoms or signs in the elderly population dizziness may arise in association with central nervous system diseases, such as vertebrobasilar insufficiency and multiple sclerosis. These diseases can affect the peripheral vestibular system, resulting in concomitant labyrinthine symptoms and signs.^34^

Regarding the number of diseases affecting the elderly, we found a mean of 4.5 diseases, with 48.9% reporting five or more associated diseases. Although it lacked a significant statistical correlation, we found that 93.8% of elderly patients with dizziness have three or more associated diseases. A survey of elderly residents in the city of Cuiabá (MT) revealed the presence of three or more diseases in 42.0% of the subjects with dizziness.^21^ In another study consisting of institutionalized elderly subjects, this occurrence indicated a 7.3 times greater chance of having an episode of dizziness, compared to those who had no diseases at all.^35^

Although more elderly subjects took medications for the nervous system (89.6%), the digestive system (83.6%) and the cardiovascular system (68.3%), those taking medicines for the muscular system (57.3%) were statistically significantly more likely to experience dizziness.

A study conducted in Finland revealed that institutionalized elderly subjects without dizziness took an average of 7.9 drugs per person, indicating that polypharmacy in LSIEs is still a current problem.^36^ In the present study, we found that our population used a mean of 7.8 medications per elderly person, with a maximum number of 17 drugs, as well as a high incidence (83.3%) of subjects taking 5 or more medications. This finding reflects the international literature, in which 74% of the elderly were in a state of polypharmacy, defined as taking more than five medications per patient.^37^

The prevalence of polypharmacy is high in various health sectors, with average numbers of 9.9–13.6 medicines taken by hospitalized patients; up to seven for patients in the intensive care unit, and 7.2–8.1 medications per institutionalized patient.^15^ According to the literature, the higher the consumption of drugs by the elderly such as antipsychotics, sedatives, antidepressants, antihypertensives, analgesics and anxiolytics, the greater the possibility of potential interactions and side effects of drugs, Almost all are associated with vertigo, imbalance and episodes of falls.^30^

In this study, despite the lack of significant association between clinical-functional characteristics of institutionalized elderly persons and occurrence of dizziness, there was a decreasing prevalence of 89.6%, 87.5% and 81.2% for depression, anxiety and falls, respectively, showing a clinically important relationship.

As for the depression associated with dizziness in institutionalized elderly people, in this study a prevalence of 89.6% was observed; this data contrasts with a survey which found a prevalence of 37.3% among elderly subjects in the community.^21^ Elderly patients with a higher number of depressive symptoms are more likely to have dizziness compared to those with fewer symptoms; in addition, they also tend to experience the following consequences: decreased functional performance in daily activities, anxiety and insecurity, and in the course of time these individuals may have mood changes, thus worsening depression.^4^

With regard to falls, the present study found high prevalence, differing from other studies published that reported a prevalence of 50.0%,^19^ 62.3%^26^ and 14.9%.^38^

When asked about their self-perceived sight and hearing, 35.4% and 37.5% of the elderly with dizziness, respectively, chose a “poor” option. In another study involving community-based elders with dizziness, an occurrence of visual (100%) and hearing (41%) impairments was identified.^21^

Elderly persons tend to show involvement of other systems related to vestibular function, including hearing and visual impairment.^28^ Hearing impairment and presence of vertigo are conditions resulting from the high sensitivity of auditory and vestibular systems to common clinical problems in the process of aging. These individuals commonly indicate that their visual system also suffers the effects of advancing age, and ocular changes, such as cataracts and glaucoma, that are responsible for a decrease in visual acuity, and which contributes to static and dynamic instability of the body.^2^

The main consequence of the natural aging process of the vestibular system is a degeneration of the vestibulo-ocular reflex (VOR),^39^ the result of which is instability with body rotation and, consequently, a deviation when walking.^40^ With its origin in the semicircular canals, VOR operates by generating eye movements at the same speed as head movement, but in the opposite direction,^41^ in order to maintain a stable visual field during head movement,^42^ thus allowing a crisp, clean image even during movement.^41^

Regarding dizziness complaints, the population of elderly patients in our study reported that their episodes began more than one year ago (58.5%). Our data differs from that obtained in the study by Souza,^22^ that showed that 54.0% reported an onset occurring five or more years ago, thus demonstrating the chronic nature of dizziness in that population.

In our study, there was a predominance of non-rotational dizziness, defined as an imbalance, in 54.1% of the elderly subjects, similar to the result obtained by Ferreira and Yoshitome,^38^ who found a prevalence of 46.2%. However, our result differs from that of Bittar's,^20^ who reported rotational dizziness in 38.0% of the elderly population in the city of São Paulo.

The triggering factors for dizziness more often reported by the elderly in this study were turning the head from side to side (39.7%) and lifting the trunk quickly (35.4%). Some authors state that postural hypotension is associated with an increased risk of dizziness and falls.^28^, ^38^ It is common for older people to report various types of movements being associated with dizziness. Studies show that up to 74% of older people reported more than one triggering activity resulting in dizziness,^4^, ^30^ with the most frequently cited being rising from a decubitus position (range of 45.9–58.0%)^28^ and turning the head (range of 25.8–67.5%).^24^ Keeping the head still in any position was the most commonly reported triggering factor for dizziness; this was identified in 60% of the elderly.^22^ Such head movements or positions promote excitation of the vestibular system and often cause vertigo and other types of dizziness, as in the cases of benign paroxysmal positional vertigo (BPPV) prevalent in older patients.^28^

Short-term dizziness was present in 57.4% of the institutionalized elderly participants, in line with the studies of Gassmann and Rupprecht^30^ and of Souza,^22^ in which the prevalence was 48.9% and 44%, respectively. For those elderly who reported long-term dizziness this can be explained by the difficulty in obtaining a full vestibular compensation by the aging population.^28^

When asked about the frequency of dizziness, 54.2% of the elderly in our study reported an occurrence of episodes of more than once a month, which agrees with the findings of Souza,^22^ who found a sporadic frequency of dizziness in 66% of elderly subjects. That author also reported that a regular and constant occurrence of dizziness is usually seen in older people with a more pronounced and disabling clinical type of dizziness.

Regarding self-perception of dizziness intensity as measured by EVA, in the current study an average of 6.2 points (±1.1) was reported (minimum and maximum scores: 3 and 8 points, respectively), similar to the findings of a study with elderly people with vestibular dysfunction that showed a mean of 6.62 points (±2.45) with minimum and maximum scores of zero and 10, respectively.^28^ Our male subjects also showed a significantly stronger intensity of dizziness (EVA) when compared to women. However, we could not find any studies investigating this association to compare with our findings.

EVA is the most used tool for assessing the subjective perception of patients with respect to the degree or intensity of dizziness,^43^ postural instability,^44^ and/or body imbalance.^40^, ^45^

In our study, the daily activity most impaired was physical activity (87.5%). A study including active and sedentary elderly subjects from the community noted that the group of sedentary seniors (33%) has more complaints of dizziness than the active ones (20%).^46^

A prevalence of neuro-vegetative symptoms in association with dizziness was reported by 52.1% of the elderly subjects, which agrees with studies showing an association between dizziness and neuro-vegetative symptoms and psychological changes.^3^, ^28^ In this study, the most commonly reported eating habit was coffee consumption (87.5%). One study suggests that certain eating habits such as the consumption of coffee, mate tea (“chimarrão”) and sugar, as well as smoking, among others, should be avoided by elderly patients with balance disturbances, as these habits may exacerbate cochleovestibular symptoms, making the vestibular compensation even slower.^47^

In the current study, DHI functional domain indices are significantly higher for older people who need walking aids, suffer falls, and for those who show anxiety, a finding which agrees with a study that evaluated elderly patients with chronic vestibular disease and observed a significant association between falls and functional DHI (*p* = 0.010).^48^ According to Peres and Silveira,^49^ the functional domain of DHI is important to the quality of life of seniors, since it interferes with the individual's dependence on the social context.

Scherer, Lisboa and Pasqualotti^5^ identified a significant difference (*p* = 0.02) in the functional aspect for complaints of body imbalance. Their findings differ from studies that investigated the most impaired functional domain in elderly patients with dizziness, with means of 16.80 and 8.60 points, respectively.^48^, ^50^

In the current study, we could identify a directly proportional, but not significant, relationship between elderly's age and total DHI score; that is, for older seniors, a higher total DHI score was found. This finding is in line with a study that observed greater commitment of functional aspects with increasing age, but did not find the same relationship between total DHI score and the patient's age.^10^

Changes in balance resulting from dizziness trigger a series of psychosocial consequences that are manifested through negative feelings, interfering with activities of daily life of the affected individuals.^11^, ^34^

In view of these considerations, this study makes clear the need for interventions for the elderly in long-stay institutions, aiming at prevention, promotion and possible rehabilitation of the health of institutionalized elderly persons, with the objective of reducing the incidence of falls and the resulting consequences of such accidents.

## Conclusions

The typical elderly subject in our study of subjects with dizziness living in long-stay institutions was an unmarried Caucasian women aged over 70 years, who did not complete an elementary level of education and who had been institutionalized less than five years. She had multiple other comorbidities, took multiple mediations, experienced a mood disorder, and was undergoing psychological treatment. As a group, our subjects indicated that they had poor visual and auditory acuity, and experienced imbalance episodes followed by short-duration vertigo, that had been present for years, occurred more than once a month, were triggered by head and body movements and mainly affected functional aspects.

## Conflicts of interest

The authors declare no conflicts of interest.
